# Estimation and application of population attributable fraction in ecological studies

**DOI:** 10.1186/s12940-019-0492-4

**Published:** 2019-06-13

**Authors:** Cheng-Kuan Lin, Szu-Ta Chen

**Affiliations:** 1 Department of Environmental Health, Harvard T.H. Chan School of Public Health, 665 Huntington Avenue, Boston, MA 02115 USA; 2 Department of Epidemiology, Harvard T.H. Chan School of Public Health, 667 Huntington Avenue, Boston, MA 02115 USA

**Keywords:** Population attributable fraction, Ecological study

## Abstract

Estimation of population attributable fraction (PAF) requires unbiased relative risk (RR) by using either Levin’s or Miettinen’s formula, on which decision depends on the available exposure information in reference group, not the types of studies. For ecological studies and studies with aggregated outcomes, once having unbiased RRs, Levin’s and Miettinen’s formulae would provide identical PAF estimates. PAF could also be applied to compare relative burdens of disease between countries across time, which is an additional information in consideration of country-level policies.

To the Editor

Population attributable fraction (PAF) is widely used to measure the disease burden attributable to a given risk factor. Concerns on improper estimation of PAF in ecological studies are raised [1] in consideration of potential ecological bias. However, if unbiased relative risks (RR) are available, estimation of PAF from ecological studies is feasible, either by using Levin’s or Miettinen’s formula.

In a recent published ecological study [2], for example, the PAF is referred to the proportion of subjects with lung cancer that would have not occurred if coal-fired power plants (the exposure of interest) were absent counterfactually, assuming the same probability of getting lung cancer in the exposed and unexposed groups with the remaining risk factors. Considering a randomly selected country *i*, the numbers of subjects with and without lung cancer in the exposed and unexposed groups can be presented as Table 1.

**Table 1:** Numbers of subjects with and without disease in exposed and non-exposed groups

	With disease (Cases, lung cancer)	Without disease (reference)	Total
Exposed group	a	b	X
Unexposed group	c	d	Y

$$ \mathrm{PAF}=\frac{O_i-{E}_i}{O_i}=\frac{\left(a+c\right)-\left(X\frac{c}{Y}+c\right)}{a+c}=\frac{a-X\frac{c}{Y}}{a+c} $$

where O = observed numbers of subjects with lung cancer; E = expected numbers of subjects with lung cancer had everyone not been exposed in country *i*.

PAF could also be expressed as either Levin’s or Miettinen’s formula, respectively.

$$ \mathrm{PAF}=\frac{P_e\times \left( RR-1\right)}{P_e\times \left( RR-1\right)+1}\kern0.5em $$(Levin’s formula) [3].

$$ ={P}_c\times \frac{RR-1}{RR} $$ (Miettinen’s formula) [4].

where.

${P}_e=\frac{X}{X+Y}$, the proportion of subjects being exposed in the population;

$\mathrm{RR}=\frac{\frac{a}{X}}{\frac{c}{Y}}=\frac{a\times Y}{X\times c}$, relative risk of lung cancer comparing the subjects among exposed and unexposed groups;

${P}_c=\frac{a}{a+c}$, the proportion of exposed cases among lung cancer subjects.

Mathematically, the PAF calculated by using Levin’s formula would be identical to that from Miettinen’s formula, regardless whether *P*_*e*_ = *P*_*c*_ = 1 or not, as proved below.

Levin’s formula:

$$ \mathrm{PAF}=\frac{P_e\times \left( RR-1\right)}{P_e\times \left( RR-1\right)+1}=\frac{\frac{X}{\left(X+Y\right)}\times \frac{\left( aY- Xc\right)}{Xc}}{\frac{X}{\left(X+Y\right)}\times \frac{\left( aY- Xc\right)}{Xc}+1}=\frac{\frac{XaY-{X}^2c}{\left(X+Y\right) Xc}}{\frac{X\left( aY- Xc\right)+\left(X+Y\right)c}{\left(X+Y\right)c}}=\frac{X\left( aY- Xc\right)}{X\left( aY- Xc+ Xc+ Yc\right)}=\frac{aY- Xc}{aY+ cY}=\frac{a-X\frac{c}{Y}}{a+c} $$

Miettinen’s formula:

$$ \mathrm{PAF}={P}_c\times \frac{RR-1}{RR}=\frac{\left(\frac{a}{a+c}\right)\times \left(\frac{aY- Xc}{Xc}\right)}{\frac{aY}{Xc}}=\frac{aY- Xc}{aY+ cY}=\frac{a-X\frac{c}{Y}}{a+c} $$

One major reason that researchers commonly use Levin’s formula to estimate PAF in case-control studies and use Miettinen’s formula in cohort studies, respectively, is because of data availability. In case-control studies, researchers have sufficient information of the four cells in Table 1. Whereas in cohort studies and studies with aggregated data (e.g., standardized incidence ratio (SIR) and standardized mortality ratio (SMR) studies), most researchers do not have the exposure information among subjects without diseases (i.e. b and d cells in Table 1), and thus *P*_*e*_ is not available. Once having sufficient information in observational studies, the use of either Miettinen’s or Levin’s formula would yield an identical PAF estimate.

Furthermore, although both requiring unbiased RRs for estimation of PAF in Levin’s and Miettinen’s formulae, methods to obtain the unbiased RRs are different according to study types, such as using confounding adjustment (mostly from regression models) in case-control studies and stratification (e.g., SMR stratified by age and sex) in observational studies [5]. Theoretically, unbiased RRs from either fully adjustment (i.e., no residual confounding) or fully stratification (i.e., no residual confounding within stratum) should be identical and feasible for PAF estimation [6]. World Health Organization and Institute for Health Metrics and Evaluation applied a hybrid method, which age- and sex-stratified RRs were retrieved by prior meta-analysis or regression models, and summarized in standardized populations to estimate PAFs (i.e., global burden of diseases) [7]. Given fully adjusted and unbiased estimation, RRs derived from ecological studies and other studies with aggregated outcomes would be as valid as those from case-control studies, and therefore, are legit for PAF estimation.

Lastly, PAF estimates could be interpreted as relative strength of a relationship between exposure and disease, regardless of the nature of association or causation [8], and subsequently be applied to compare relative burden of diseases across countries/populations. In Lin’s study [2], for example, relative burden of lung cancer between countries across time contribute valuable information in consideration of country-level policies.

In conclusion, valid PAF estimation could be achieved from both Levin’s and Miettinen’s formulae with sufficient information in different types of studies with unbiased RR estimation, regardless stratification or adjustment. Comparison of PAFs between countries across time might provide additional information, along with the point estimate per se.

## Data Availability

Not applicable.

## Abbreviations

PAF: Population attributable fraction
RR: Relative risks
SIR: Standardized incidence ratio
SMR: Standardized mortality ratio
